# Clinical features, myocardial injury and systolic impairment in acute myocarditis

**DOI:** 10.1136/openhrt-2024-002901

**Published:** 2024-12-03

**Authors:** Vijay Shyam-Sundar, Greg Slabaugh, Saidi A Mohiddin, Steffen Erhard Petersen, Nay Aung

**Affiliations:** 1 Advanced Cardiovascular Imaging, Queen Mary University of London William Harvey Research Institute, London, UK; 2 Department of Cardiology, Barts Heart Centre, St Bartholomew's Hospital, London, UK; 3 Digital Environment Research Institute, Queen Mary University of London, London, UK; 4 William Harvey Research Institute, Queen Mary University of London, London, UK

**Keywords:** Biomarkers, Magnetic Resonance Imaging, Myocarditis, Diabetes Mellitus

## Abstract

**Objective:**

Cardiovascular magnetic resonance (CMR) is increasingly used in the diagnosis of myocarditis, with myocardial injury and systolic dysfunction playing key roles in the prognosis of this clinical setting. The clinical determinants of myocardial injury and systolic impairment in acute myocarditis are poorly defined. The aim of the current study is to assess the association of laboratory markers, late gadolinium enhancement (LGE) and left ventricular ejection fraction (LVEF) in patients with acute myocarditis.

**Methods:**

We completed a retrospective cohort study from a tertiary referral centre in London with CMR and acute myocarditis. Cases with cardiomyopathy were excluded. Missing data was imputed for selected clinical variables. We evaluated the association between peak troponin and LGE extent and LVEF. We adjusted the models for age, sex and time to CMR with a sensitivity analysis adjusting for body mass index and cardiovascular risk factors including hypertension, dyslipidaemia, diabetes mellitus and smoking.

**Results:**

127 patients had abnormal T2-weighted imaging/mapping results with 118 (93%) presenting with chest pain and/or shortness of breath. Left ventricular LGE was identified in 118 (93%) patients and LVEF was 58±11%. The median time from the peak troponin to CMR was 1 day (IQR 0–6 days). The highest tertile of peak troponin was associated with more LGE (incident rate ratio 1.33, 95% CI: 1.07 to 1.64) and a lower LVEF (coefficient −5.3%, 95% CI: −9.5% to −1.1%). Diabetes was also associated with more LGE (incident rate ratio 1.90, 95% CI: 1.37 to 2.61) and lower LVEF (coefficient −8.9%, 95% CI: −14.7% to −1.8%).

**Conclusions:**

Peak troponin is associated with more LGE and a lower LVEF even after accounting for demographics and comorbidities. Myocardial injury and systolic dysfunction play key roles in prognosis and future work incorporating clinical features into a risk prediction model may enable better risk stratification in acute myocarditis.

**WHAT IS ALREADY KNOWN ON THIS TOPIC**

- Myocardial injury and systolic impairment have been identified as prognostically important on cardiovascular magnetic resonance in acute myocarditis. The clinical features associated with myocardial injury and systolic impairment in acute myocarditis are poorly defined.

**WHAT THIS STUDY ADDS**

- Male sex, peak serum troponin, diabetes mellitus and smoking history are important determinants of left ventricular ejection fraction, a measure of systolic function and late gadolinium enhancement extent, a surrogate for myocardial injury.

**HOW THIS STUDY MIGHT AFFECT RESEARCH, PRACTICE OR POLICY**

- Clinical features associated with myocardial injury and systolic impairment in acute myocarditis identified in this study, including sex, diabetes, smoking and troponin, could be incorporated into a risk prediction model. This may enable better risk stratification in acute myocarditis.

## Introduction

 The diagnosis of acute myocarditis has been made more frequently due to the introduction and increased availability of high-sensitivity troponins and cardiovascular magnetic resonance (CMR) imaging. CMR is increasingly used in diagnosing myocarditis as opposed to endomyocardial biopsy, which was considered the reference standard for diagnosis. Myocarditis remains a challenging diagnosis because of heterogeneity in clinical manifestation, severity of disease presentation and in short-term and long-term outcomes. Clinical context is central to diagnosis and should be compatible with myocarditis and myocardial inflammation, which can be identified by CMR. CMR can also be used for follow-up after an episode of acute myocarditis, although its role and timing have yet to be established. When left ventricular ejection fraction (LVEF) on the initial CMR scan is preserved, the risk of subsequently developing inflammatory cardiomyopathy is very low^1^. The outcomes following acute myocarditis are varied, including spontaneous recovery, arrhythmia, persistent left ventricular (LV) dysfunction and, in some individuals, chronic inflammatory cardiomyopathy with consequent implications for monitoring and prognosis. Multiparametric CMR is useful for functional evaluation and tissue characterisation, and several prognostic imaging markers on CMR have been identified in acute myocarditis. CMR is increasingly used in diagnosing myocarditis, with myocardial injury and systolic dysfunction playing key roles in the prognosis of this clinical setting. However, the clinical determinants of myocardial injury and systolic impairment in acute myocarditis leading to poor clinical outcomes are not well defined.

We describe the clinical presentation and characteristics of a retrospective cohort of acute myocarditis from a large tertiary referral centre in London, all of whom had completed CMR studies. The aim of the current study is to assess the association of laboratory markers, late gadolinium enhancement (LGE) and LVEF in patients with acute myocarditis.

## Methods

### Defining acute myocarditis

CMR reports of all patients enrolled in the biorepository of a tertiary referral centre from 30 January 2015 to 15 August 2023 were interrogated with a natural language processing algorithm to identify individuals with elevated myocardial T2 signal, indicating myocardial oedema. Acute myocarditis was defined as symptoms and clinical findings consistent with recent myocardial damage alongside the suggestion of myocardial oedema on CMR. Reports and electronic health records were reviewed, and cases not in keeping with a clinical definition of acute myocarditis and those with a known diagnosis of an inflammatory or non-inflammatory form of cardiomyopathy were excluded. Baseline demographic, clinical, laboratory and imaging parameters were collated. CMR outcomes included LVEF and LGE, with the extent of LGE quantified by the number of affected LV segments in a 17-segment model.

### Dealing with missing data

Missingness for selected variables is shown in table 1. We performed imputation for both continuous and categorical variables in the data set. Imputation of missing categorical and continuous data was performed using the MissForest package in Python.^2^ Other imputation methods were considered, including multiple imputation chained equations, K nearest neighbour and simple imputation strategies (mean, median, mode). MissForest was chosen for this study as it outperforms other methods in terms of imputation error and can maintain predictive ability using imputed values in clinical predictive models.^3^

**Table 1:** Baseline characteristics

		Missing	Overall	LVEF <55%	LVEF ≥55%
**n**			127	40	87
Age, years, mean (SD)		0	41.2 (16.4)	40.1 (16.6)	41.7 (16.4)
Sex, n (%)	Female	0	46 (36.2)	11 (27.5)	35 (40.2)
	Male		81 (63.8)	29 (72.5)	52 (59.8)
Ethnicity, n (%)	Not disclosed	7			
	White		59 (49.2)	19 (50)	40 (48.8)
	Black		12(10)	6 (15.8)	6 (7.3)
	Asian		15 (12.5)	6 (15.8)	9 (11.0)
	Other		34 (28.3)	7 (18.4)	27 (32.9)
BMI, kg/m^2^, median (Q1, Q3)		0	25.0 (22.0, 28.4)	25.0 (22.0, 28.0)	25.1 (22.5, 28.9)
Diabetes, n (%)	No	15	98 (87.5)	33 (82.5)	65 (90.3)
	Yes		14 (12.5)	7 (17.5)	7 (19.7)
Smoking status, n (%)	Current smoker	32	32 (33.7)	13 (39.4)	19 (30.6)
	Ex-smoker		12 (12.6)	5 (15.2)	7 (11.3)
	Non-smoker		51 (53.7)	15 (45.5)	36 (58.1)
Hypertension, n (%)	No	14	88 (77.9)	31 (77.5)	57 (78.1)
	Yes		25 (22.1)	9 (22.5)	16 (21.9)
Dyslipidaemia, n (%)	No	13	93 (81.6)	33 (82.5)	60 (81.1)
	Yes		21 (18.4)	7 (17.5)	14 (18.9)
Peak troponin, ng/L, median (Q1, Q3)		38	880.0 (285.0, 1585.0)	1230.5 (736.8, 2388.5)	672.0 (266.0, 1123.0)
C-reactive protein, mg/L, median (Q1, Q3)		40	35.0 (7.0, 96.5)	66.0 (17.0, 127.5)	23.0 (5.5, 83.0)
Creatinine, μmol/L, mean (SD)		15	78.1 (22.7)	79.9 (19.0)	77.2 (24.5)
Platelet, count x 10^9^, mean (SD)		33	273.4 (102.0)	266.3 (99.1)	278.2 (104.6)
LVEF on transthoracic echocardiogram, %, median (Q1, Q3)		36	55.0 (45.0,55.0)	45.0 (35.0, 55.0)	55.0 (55.0, 55.0)
LVEF on CMR, %, mean (SD)		0	57.7 (10.6)	45.4 (7.5)	63.3 (6.2)
Number of LGE segments, median (Q1, Q3)		0	3.0 (2.0, 5.0)	5.0 (2.0, 8.0)	3.0 (2.0, 4.5)

The mean and standard deviation (SD)standard deviation (SD) isare shown for continuous variables, which are normally distributed, and median and interquartile range interquartile range (Q1; 1stfirst quartile, Q3; 3rdthird quartile) for non-normal variables. Categorical variables are shown with the number in each category, n and percentages for each category. For each variable, missing data is recorded and data is grouped by left ventricular ejection fraction (LVEF) with a cut-off.

BMI, body mass index; CMRcardiovascular magnetic resonanceLGE, late gadolinium enhancementLVEF, left ventricular ejection fraction

### Regression models

Multivariable regression analyses on an imputed data set were completed to evaluate the association of peak troponin with CMR LVEF and LGE, adjusted for age, sex and time to CMR. Peak troponin was defined as the largest value of troponin T recorded and was split into tertiles before linear regression modelling to compare the effect of higher tertiles with the lowest tertile on outcomes. For LGE, a Poisson model was used to reflect the data set (ie, count data). For LVEF, a Gaussian regression model was used. A sensitivity analysis was performed additionally adjusting for body mass index (BMI) and cardiovascular risk factors including hypertension, dyslipidaemia, diabetes mellitus and smoking. All analyses were completed in Python v.3.9 with numpy, statsmodels, seaborn and matplotlib libraries used for statistical analyses.

### Patient and public involvement

The patients in this study have been enrolled in the Barts BioResource. Patient and public involvement and engagement are central to the ongoing activities of the Barts BioResource. The patient and public advisory group was involved right from the outset when the idea came about to establish the Barts BioResource to support recruitment, provide feedback on access applications and suggest amendments to the protocol and dissemination activities.

## Results

**Figure 1:**
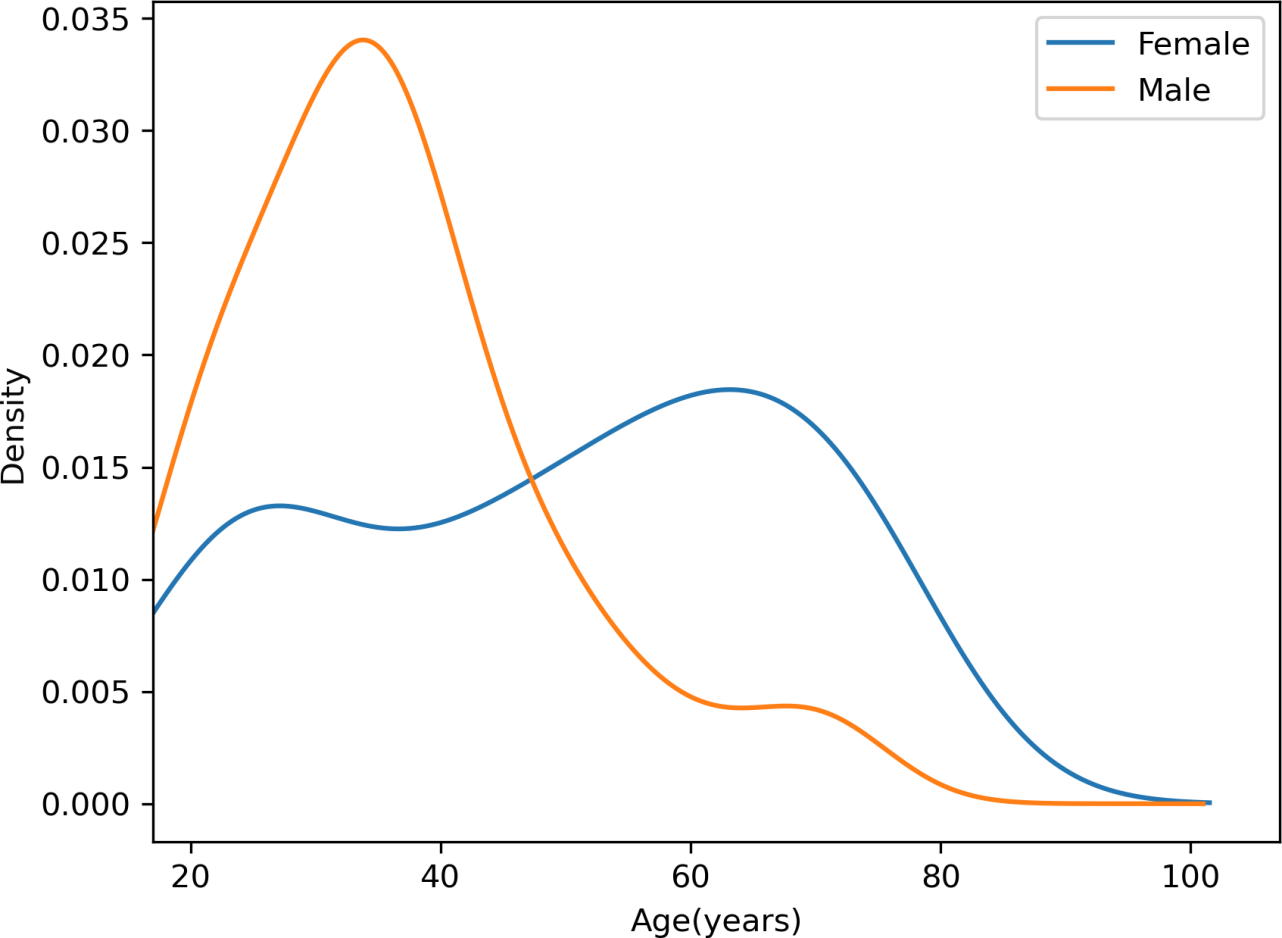
Age sex distribution for acute myocarditis. This kernel density estimate plot shows the distribution of age for males and females in our data set. On the y-axis, the density represents how frequently ages occur in each group. The blue line represents the age distribution for females and the orange line represents the age distribution for males. The male age distribution has a single peak in comparison to the female distribution which has two peaks at 24 and 73.

The baseline data for this cohort is summarised in table 1. A total of 127 patients with abnormal T2-weighted imaging and T2 mapping results and acute myocarditis were identified, of whom 118 (93%) had a presentation with chest pain and/or shortness of breath, but only 21 (18%) of this group had a preceding viral syndrome. Clinical signs of heart failure were detected in 24 (21%), 59 (46%) were white, 15 (12%) were Asian, 12 (9%) were black, 34 (27%) had other ethnicities and 7 (6%) did not disclose ethnicity. Age ranged from 17 to 75 years, with a bimodal age distribution for women (the modes were 24 and 73 years); 81 (64%) were male (mean age 36 years) (figure 1). The breakdown of cardiovascular risk factors in this cohort, including smoking, diabetes mellitus, dyslipidaemia and hypertension, is shown in table 1. The median BMI of the cohort is 25 kg/m^2^ (interquartile range (IQR) 22–28.4 kg/m^2^).

LVEF was 58±11%. The median time from the peak troponin to CMR was 1 day (IQR 0–6 days). LGE was found in 118 (93%) patients; this had a biventricular distribution in 5 (4%), and a non-ischaemic pattern in 116 (98%). Of note, the two patients with ischaemic pattern LGE also had systemic autoimmune disease.

Missingness was noted in the data set, with data deemed missing at random (table 1). Following imputation, the sample size available for regression modelling was 127.

**Figure 2:**
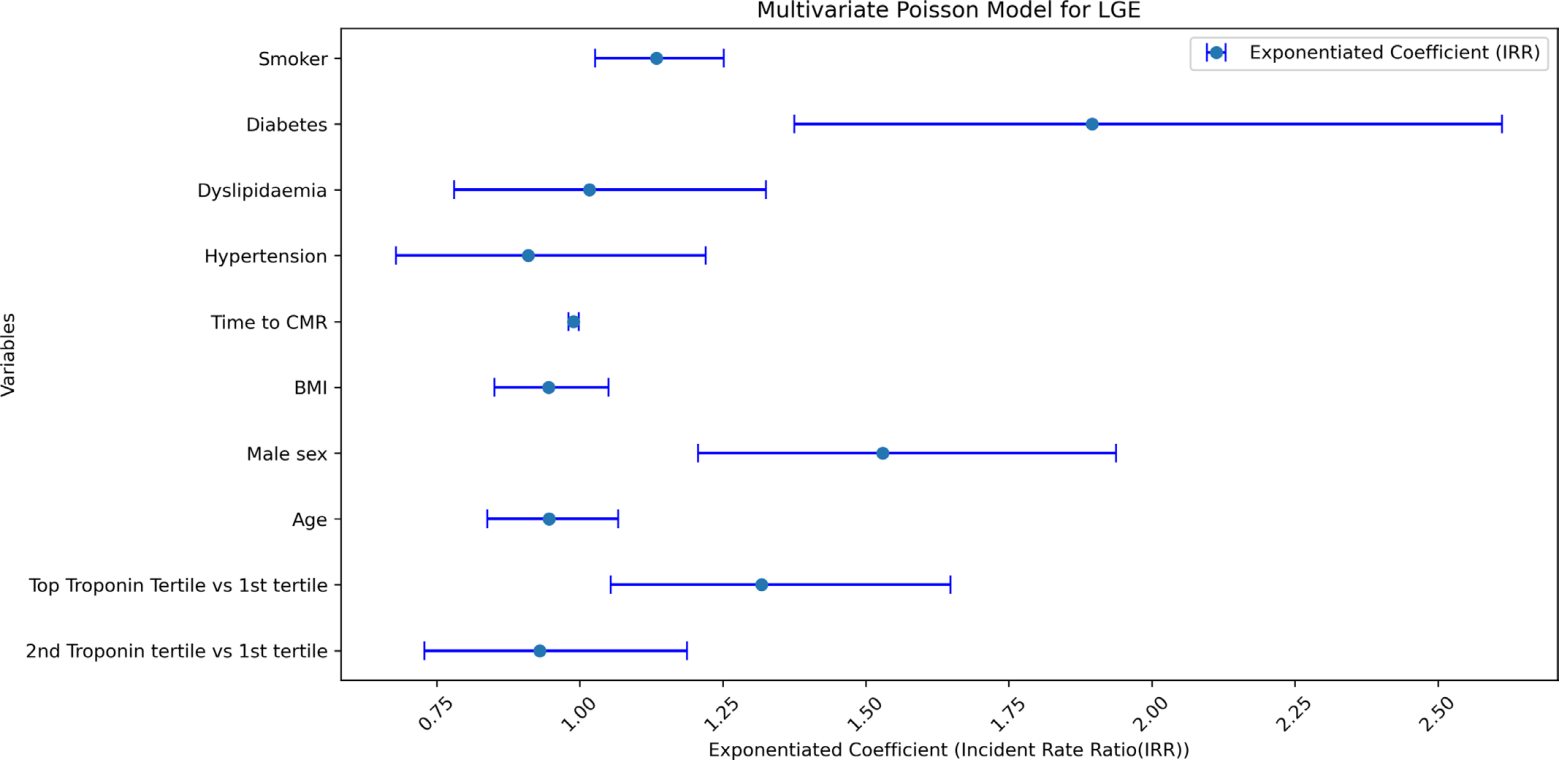
Effect size plot for multivariable Poisson Model. This plot shows the effect of individual variables (y-axis) on late gadolinium enhancement (LGE) extent in the regression model with coefficients and 95% CIs displayed on the x-axis. The coefficients have been exponentiated for easier interpretation. Top troponin tertile, diabetes and male sex are all significantly associated with increased numbers of left ventricular segments with LGE. IRR, Incident Rate Ratio. CI, confidence interval ; IRR, incident rate ratio.

In a minimally adjusted model, the highest tertile of peak troponin was associated with greater LGE (incident rate ratio 1.38, 95% CI: 1.11 to 1.73, p=0.004), which corresponded to 38% more LV segments with LGE for those with the highest tertile of troponin compared with the lowest. After adjusting for BMI and risk factors for atherosclerosis, the association between the highest tertile of peak troponin and LGE was mildly attenuated (incident rate ratio 1.33, 95% CI: 1.07 to 1.64, p=0.01), reflecting 33% more LV segments with LGE for those with the highest troponin values compared with those in the lowest tertile of troponin (figure 2). In both models, male sex is significantly associated with a greater LGE extent, in keeping with other studies in acute myocarditis where sex-specific differences in LGE are reported.^4^ Additionally, in the model adjusted for BMI and risk factors for atherosclerosis, diabetes mellitus was significantly associated with the number of affected LGE segments (figure 2). The interaction between diabetes mellitus and troponin was investigated further. The interaction term between diabetes mellitus and troponin in the fully adjusted model for LGE was statistically significant (incident rate ratio 2.27, 95% CI: 1.58 to 3.27, p<8.97×10^−6^). This suggests that the association between elevated troponin and greater LGE is stronger in people with diabetes than in those without diabetes.

**Figure 3:**
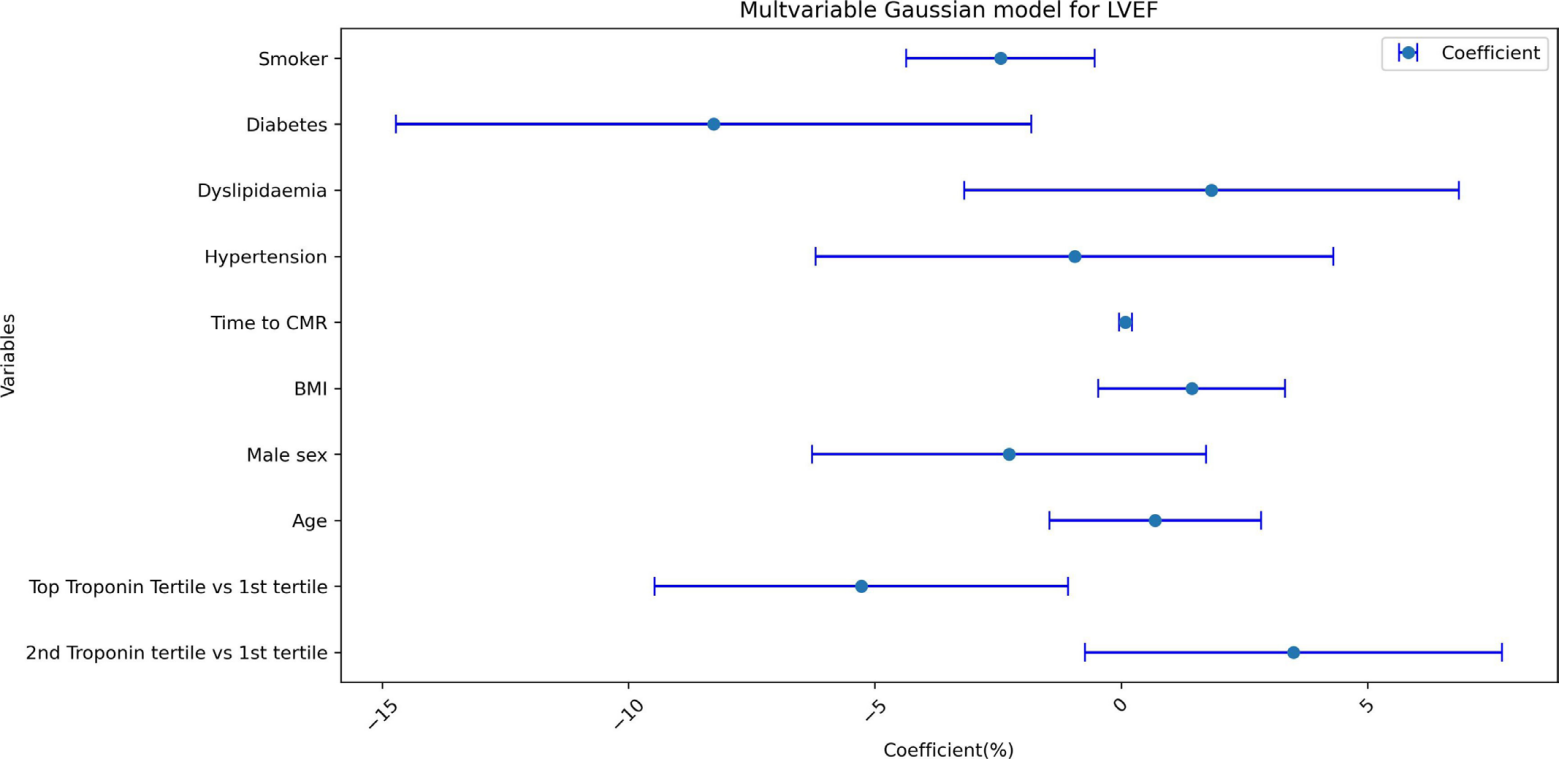
Effect size plot for multivariable Gaussian model for LVEF. This plot shows the effect of individual variables on left ventricular ejection fraction (LVEF) in the regression model with coefficients and 95% CIs displayed on the x-axis. Diabetes, smoking and top troponin tertile are independently and significantly associated with a reduced LVEF. BMI, body mass index; CI, confidence interval; CMR, cardiovascular magnetic resonance.

In a minimally adjusted model, the highest tertile of peak troponin was associated with a lower LVEF (coefficient −5.1%, 95% CI: −9.5% to −0.7%, p=0.02). After adjusting for BMI and cardiovascular risk factors, the highest tertile of peak troponin remained associated with a lower LVEF with no attenuation of effect size (coefficient −5.3%, 95% CI: −9.5% to −1.1%, p=0.01) (figure 3). In addition to troponin, diabetes and smoking were both also associated with a lower LVEF; these associations were independent of age and sex (figure 3).

## Discussion

Our study highlights the association between peak troponin, a lower LVEF and greater LGE extent in acute myocarditis. We identified diabetes mellitus as a predictor of both lower LVEF and greater LGE extent in myocarditis, while smoking was associated with a lower LVEF. Additionally, male sex was independently associated with greater LGE extent.

### Age/sex variation

A better understanding of sex differences in the incidences, causes, types and natural history of myocarditis is an important component in driving improvements in patient care. A higher incidence of most cardiovascular diseases, including myocarditis, is observed in men, but cardiovascular disease is the leading cause of mortality in both sexes.^5^ It is known that atypical symptoms and underdiagnosis of cardiovascular disease may be a cause for the under-representation of women in cohorts of myocardial infarction.^6^ In completed cardiovascular trials, women tend to be under-represented.^7^ This has led to calls for sex-specific reporting of trial data and guidance for specific cardiovascular diseases.

In this study, we identified a greater proportion of men than women with myocarditis, with a sex ratio of 1:1.8 (female:male).^8 9^ The mean age at presentation for men was 36 years, concordant with the known preponderance of myocarditis in younger men (mean 36 years) as published in other cohorts.^10 11^ In contrast, the mean age at presentation for women was 50 years. This is consistent with the data from studies reporting age and sex that myocarditis is far more frequently observed to occur in men under the age of 50; after this age (or after menopause), the incidence in women may be more equal to that in men.^9^

In this study, male sex is an independent predictor of the number of LV segments with LGE in the multivariable regression model. This may mean that male patients with myocarditis have greater myocardial injury, which may represent a different type of myocarditis. Men are also thought to be at increased risk of developing heart failure from myocarditis.^12^ Therefore, men may require closer follow-up after index presentation with acute myocarditis. Limited data systematically investigating the impact of male sex on LGE extent has been published. One cohort study from Basel suggested that compared with male patients, female patients had less LGE on CMR (one vs three segments).^13^

Much of our understanding of the pathogenesis of myocarditis has been developed from animal models. One hypothesis is that cardiac inflammation is strongly influenced by sex hormones and their interaction with immune cells and cardiac tissue with the immunological profile modulated by oestrogen and testosterone.^14^ In the Fairweather animal model of viral myocarditis, biological female mice with low testosterone-to-oestrogen ratio generate a robust immune response to infection, inhibiting proinflammatory responses and promoting a regulatory inflammatory response.^14^ In contrast, biological male mice with a high testosterone-to-oestrogen ratio develop a proinflammatory immune response, promoting proinflammatory and profibrotic responses without regulatory elements.^15^ There are no disease-specific therapies for myocarditis at present and understanding sex-specific effects will aid the development of precision medicine approaches to myocarditis in the future.

### Myocardial injury

Peak troponin is a sensitive serum biomarker for myocarditis and provides evidence of myocardial injury.^16^ Peak troponin was associated with LGE in our study and LGE extent on CMR has been associated with serum troponin in other studies of acute myocarditis.^17 18^

LGE represents an expansion of the interstitium, and will not always indicate irreversible myocardial injury. In the acute setting, it may also represent myocardial oedema, with complete LGE resolution on follow-up CMR in some cases of myocarditis.^19^

In our cohort, a high proportion (93%) of patients with myocarditis were observed to have LGE on CMR. The median number of LV segments with LGE was 3 (out of 17 segments). Meta-analyses have reported the association of increased LGE and reduced LVEF with worse outcomes. Still, it is unclear whether LGE has an independent prognostic role above and beyond LVEF in acute myocarditis.^20 21^ The presence of LGE increases the risk of all-cause mortality, cardiac death and sudden cardiac death in those with viral myocarditis.^22^ The greater extent of LGE has been associated with greater risks of major adverse cardiovascular events.^23^ Even in those patients with myocarditis with preserved LV systolic function, LGE on CMR is a prognostic predictor of relevant clinical endpoints such as cardiac death, hospitalisation for heart failure and resuscitated cardiac arrest.^24^ In contrast, a CMR with normal function and no evidence of LGE in myocarditis is associated with a better prognosis with fewer major adverse cardiovascular events.^24 25^

### LV systolic function

Initial deterioration in LVEF is associated with a worse clinical outcome and is predictive of lower LVEF at follow-up.^1 26^ In our cohort, LVEF was 58±11%, reflecting relatively preserved or mild LV dysfunction in many patients. Although LVEF and LGE on CMR are both prognostic, the scope for prediction of other relevant endpoints, such as incident heart failure or life‐threatening arrhythmias in patients with acute myocarditis, may be limited.

### Diabetes mellitus

In our study, diabetes mellitus (type 2) is associated with myocardial injury features, including greater LGE extent and a lower LVEF independent of age, sex, hypertension, dyslipidaemia, smoking status and BMI. In published data, diabetes mellitus does not differ by sex in myocarditis.^27^ In our data set, individuals with diabetes mellitus have an age of 61±16 years and troponin of 1224±1237 ng/L both of which are greater than the corresponding measures for the whole cohort (table 1). Diabetes mellitus may predispose to greater myocardial injury when individuals develop myocarditis. The reasons for this remain conjectural.

Diabetic cardiomyopathy is widely recognised to occur independently of other risk factors for atherosclerosis and may be a comorbidity in our data set.^28^ Diabetes mellitus is associated with both systemic and local maladaptive inflammatory responses, which lead to the progression of diabetic cardiomyopathy.^29^ Monocytes/macrophages are leading players in the pathogenesis of diabetic cardiomyopathy with proinflammatory M1 polarisation increased and M2 anti-inflammatory responses inhibited in the diabetic heart.^30^ This modulation of the inflammatory response in the diabetic heart may explain the association with greater LGE extent. A lower LVEF has been reported in diabetic individuals without clinical cardiovascular disease in the Multi-Ethnic Study of Atherosclerosis.^31^ We postulate that diabetes mellitus itself may be associated with the presence of non-ischaemic LGE, and there is data to support this finding in patients with type 2 diabetes mellitus with CMR data.^32^

### Challenges and limitations

We acknowledge that our cohort was from a retrospectively collected data set with CMR imaging. This approach may underestimate the true incidence of acute myocarditis as affected individuals may not have had CMR studies or been eligible for CMR due to clinical instability or contraindications to CMR. Furthermore, there may be selection bias as our study used the data from a large tertiary referral centre. It may not have included individuals presenting to district general hospitals with uncomplicated myocarditis who were subsequently discharged and not referred onwards. While the sample size of 127 is relatively large for such studies, it precluded us from doing subgroup analysis to investigate the age-specific, sex-specific or ethnicity-specific effects.

For troponin T, we used the highest recorded value rather than the area under the curve due to limitations with our data set. The highest available troponin value is straightforward and simple to interpret but may miss the true peak. For this study, LGE extent was assessed by counting the number of segments where LGE was present which was simple and fast rather than a quantitative approach evaluating the total mass or percentage of LGE across the whole myocardium. Counting the number of segments is prone to subjectivity, reduced precision and may not provide reproducible and detailed information.

Our study focuses heavily on imaging endpoints. The lack of clinical follow-up outcomes such as mortality, sustained ventricular arrhythmia and hospitalisation due to heart failure limits our understanding of how these findings translate into clinical practice and relate to the long-term outlook for patients with acute myocarditis.

Lastly, some variables have incomplete data with varying degrees of missingness, as shown in table 1. This includes 38% missing values for serum troponin. We attempted to circumvent this problem by performing imputation using a random forest method in line with similar techniques used in other clinical data sets. Coronary angiography data was incomplete across this cohort but no significant ischaemic events were reported in this cohort.

### Implications and future work

Our study highlighted that, in acute myocarditis, serum troponin is independently associated with CMR markers that are themselves predictive of poorer clinical outcomes. These include reduced LVEF and greater amounts of myocardial LGE. This information could potentially be useful in clinical practice to enable risk stratification in acute myocarditis and identify people who will need close monitoring or further assessment with CMR. In-depth tissue characterisation with parametric mapping techniques (T1 and T2) may also enable image-led risk stratification in future studies.

Further assessment of adverse functional remodelling on CMR could include global longitudinal strain (GLS). GLS is an independent prognostic indicator associated with adverse outcomes even in those with normal LV systolic ejection fraction. It may improve the risk stratification of those with acute myocarditis.^33^

We also showed in this study that in addition to troponin, male sex and diabetes mellitus determined LVEF and LGE extent in acute myocarditis. This knowledge could be useful in developing an integrated clinical risk score and imaging biomarkers to predict the future risk of deleterious events, including heart failure admission and arrhythmia.

## Conclusion

We identified male sex, peak serum troponin, diabetes mellitus and smoking history as important determinants of LVEF and LGE extent in myocarditis. Our data set had a bimodal distribution of age for females, in contrast to the unimodal distribution for males. Diabetes mellitus may modulate the inflammatory response in the heart in myocarditis. Future work incorporating these clinical features into a risk prediction model may enable better risk stratification in acute myocarditis.

## Data Availability

All data relevant to the study are included in the article or uploaded as supplementary information.
